# NO gas sensor based on ZnGa_2_O_4_ epilayer grown by metalorganic chemical vapor deposition

**DOI:** 10.1038/s41598-019-43752-z

**Published:** 2019-05-16

**Authors:** Min-Ru Wu, Wei-Zhong Li, Chun-Yi Tung, Chiung-Yi Huang, Yi-Hung Chiang, Po-Liang Liu, Ray-Hua Horng

**Affiliations:** 1 Institute of Electronics, National Chiao Tung University, 1001 University Rd., Hsinchu, 30010 Taiwan Republic of China; 2 Graduate Institute of Precision Engineering, Chung Hsing University, 145 Xingda Rd., Taichung, 40227 Taiwan Republic of China; 3 Center for Emergent Functional Matter Science, National Chiao Tung University, Hsinchu, 30010 Taiwan Republic of China

**Keywords:** Environmental impact, Materials for devices

## Abstract

A gas sensor based on a ZnGa_2_O_4_(ZGO) thin film grown by metalorganic chemical vapor deposition operated under the different temperature from 25 °C to 300 °C is investigated in this study. This sensor shows great sensing properties at 300 °C. The sensitivity of this sensor is 22.21 as exposed to 6.25 ppm of NO and its response time is 57 s. Besides that, the sensitivities are 1.18, 1.27, 1.06, and 1.00 when exposed to NO_2_(500 ppb), SO_2_ (125 ppm), CO (125 ppm), and CO_2_ (1500 ppm), respectively. These results imply that the ZGO gas sensor not only has high sensitivity, but also has great selectivity for NO gas. Moreover, the obtained results suggest that ZGO sensors are suitable for the internet of things(IOT) applications.

## Introduction

Recently, gas sensors have been developed and applied in environmental monitoring, human security, medical applications, and automobiles applications^1–4^. Among those different target gases monitoring, the detection of nitric oxide (NO) has attracted considerable interest. NO is an extremely toxic oxidizing gas with a pungent odor. It is always released by the action of nitric acid on metals, such as in metal etching and pickling. Besides, it plays an important role in a human biological process such as cardiovascular, immune systems^5–7^. Moreover, NO also affects neuron operation, which causes neurodegenerative diseases^8^. Therefore, it is very important to develop NO gas sensors.

There are various types of gas sensor including electrochemical^9–11^, optical^12,13^ and semiconductor gas sensors^14–17^. Semiconductor gas sensors have great potential for commercial application in environmental monitoring and healthcare due to the properties low cost, low power consumption, long lifetime, and the ability to operate in harsh environments.

It is well known that metal oxide semiconductors, e.g. SnO_2_ and ZnO, have been extensively studied for gas sensors applications. The corresponding sensing mechanism is resulted from the oxygen vacancy, metal vacancy and the other defects existing in the thin film^18–21^. Most of the metal oxide semiconductors were deposited by sputtering and sol-gel methods. It results that the crystal structure of thin films was amorphous or polycrystal. These suggest the defects in the thin film were not easily controlled and repeated. Although the polycrystal metal oxide semiconductors showed good sensitivity to many gases, the main issues are the poor selectivity and long response time. Recently, the wide bandgap oxide materials are attracting more attention for their use in novel devices owing to their thermally and chemically inert properties. Owing to such material properties, ZnGa_2_O_4_ (ZGO) has been demonstrated and presents very promising applications from the viewpoint of device fabrication^22–24^. ZGO is a transparent and conductive oxide material with a wide bandgap of approximately 5.2 eV, and it can be grown by metalorganic chemical vapor deposition (MOCVD) and fabricated into a photodetector for deep-ultraviolet^25–27^ and power device applications^28,29^.

Although, some NO_x_ gas sensors with nanorods, nanowires, nanosheet have been fabricated^30–34^. The nanostructure gas sensor has great sensitivity due to extremely high surface-to-volume ratios. However, most thin film NO_x_ gas sensors have difficulties in sensing ppb-level of NO^35–37^ and in gas selectivity. In this study, the ZGO epilayers grown on the sapphire substrate was successfully fabricated as a channel material for NO_x_ gas sensor. The sensitivity, selectivity, and responsivity to NO at different operating temperature will be studied in this work.

## Results and Discussion

**Figure 1:**
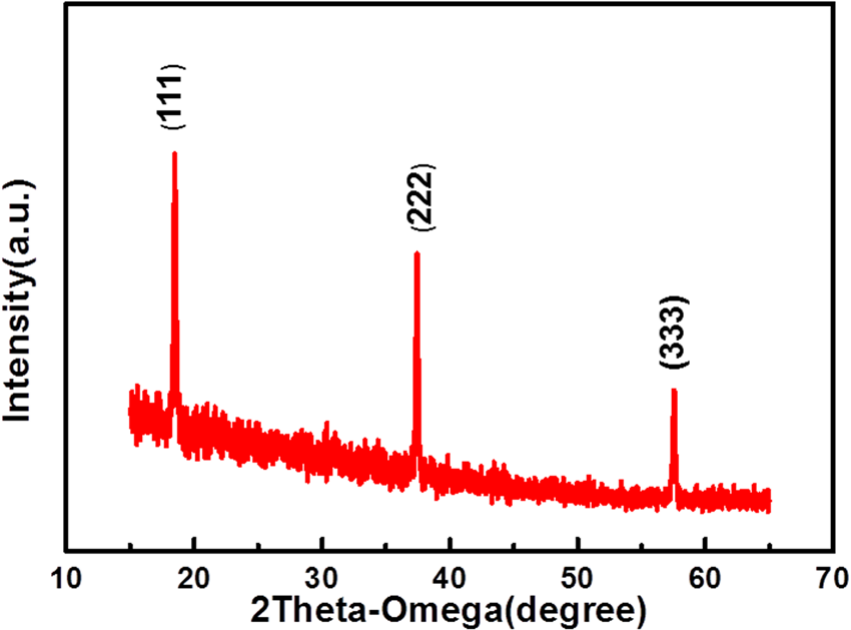
XRD pattern of ZGO thin film gas sensor.

Figure 1 shows XRD patterns of the ZGO thin film that were grown by MOCVD. The diffraction peaks about 18.40°, 37.34° and 57.49° were identified as the (111), (222), and (333) crystal plane of ZGO thin film. In general, the (333) plane was not allowed diffraction plane in the spinel crystal and the peak is always attributed to (511). Here, it can be regarded as the (333) diffraction plane due to the lattice mismatch between ZGO and sapphire. It means the ZGO eiplayer is a single crystal structure.

**Figure 2:**
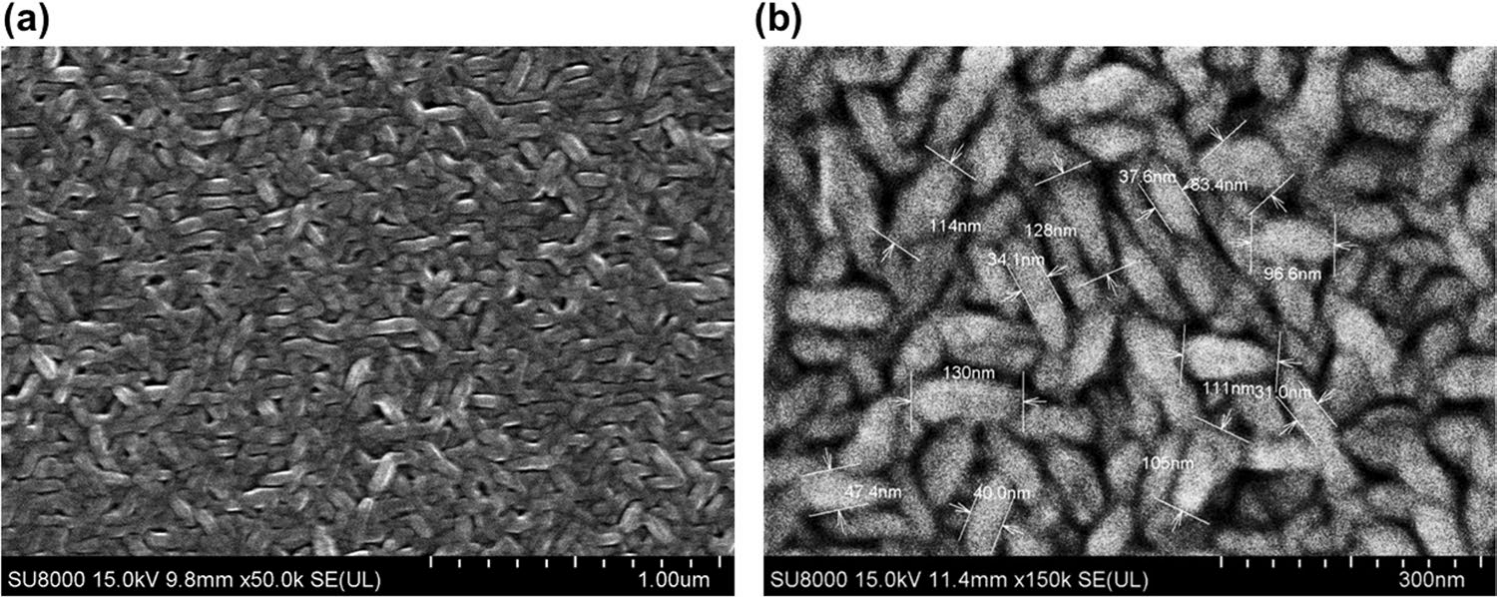
SEM micrograph of the surface of ZGO thin film gas sensor.

Figure 2(a) shows an SEM image of the ZGO thin film. It can be observed that ZGO thin film had a spindle structure. This structure offers lots of areas to react with NO gas molecules. Figure 2(b) shows the enlarged image. The length and width of spindle dimensions are about 120 nm and 40 nm, respectively.

**Figure 3:**
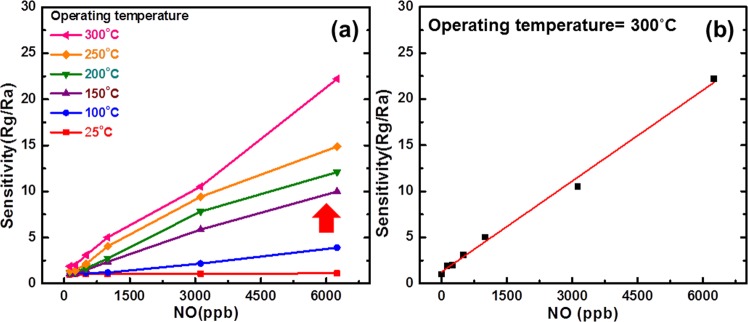
(**a**) Sensitivity of ZGO gas sensor versus different NO gas concentration at different temperatures from 25 °C to 300 °C and (**b**) Sensitivity of ZGO gas sensor as a function of NO gas concentration measured at 300 °C.

The sensor was operated at 25, 100, 150, 200, 250, 300 °C six different operating temperatures to evaluate the optimum operated temperature. Figure 3(a) illustrates the relationship between sensitivity and NO gas concentration with different operating temperature from 25 °C to 300 °C. It indicates that the sensor has the highest sensitivity at 300 °C. Figure 3(b) illustrates the sensitivity as a function of NO gas concentration as the gas sensor operated at 300 °C. The sensitivity (S) using the curve fitting has a linear relation to NO concentration (C) at 300 °C. The linear fitting denoted as

1 $${\rm{S}}=1+0.00327\times {\rm{C}}.$$

It is worthy to mention that the concentration of NO increasing to ppm level still presented a linear relationship between sensitivity and concentration.

**Figure 4:**
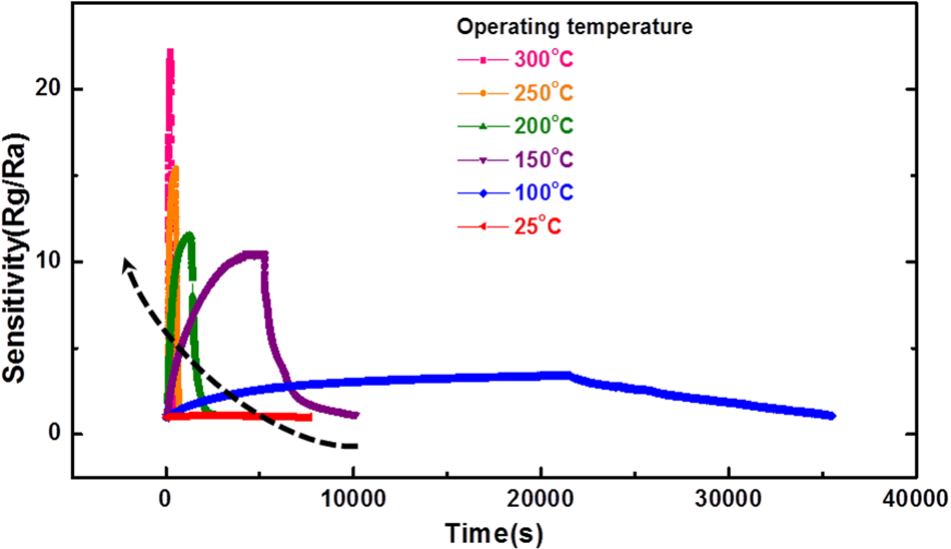
Sensitivity of ZGO gas sensor with 6250 ppb of NO at different operating temperatures from 25 °C to 300 °C.

Figure 4 shows the relationship between operating temperature and the sensitivity of ZGO gas sensor when exposed to 6250 ppb of NO. It was found that the dynamic sensitivity curve shifted to the upper left corner as operating temperature increasing (black arrow). As the temperature ramps from 25 to 300 °C, the sensitivity increases from 1.11 to 22.21. Besides of that, the response time reduces from 10053 s to 57 s and recovery time reduces from 17646 s to 78 s. In other words, the sensing properties have been extraordinarily improved after ramping operating temperature to 300 °C.

**Figure 5:**
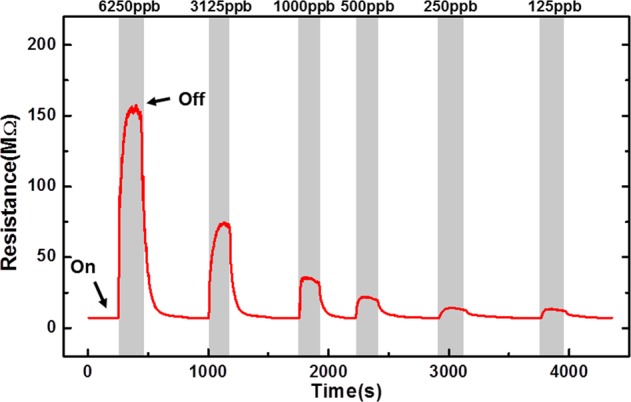
Transient response of ZGO gas sensor with six different NO gas concentrations from 125 ppb to 6250 ppb at 300 °C.

**Figure 6:**
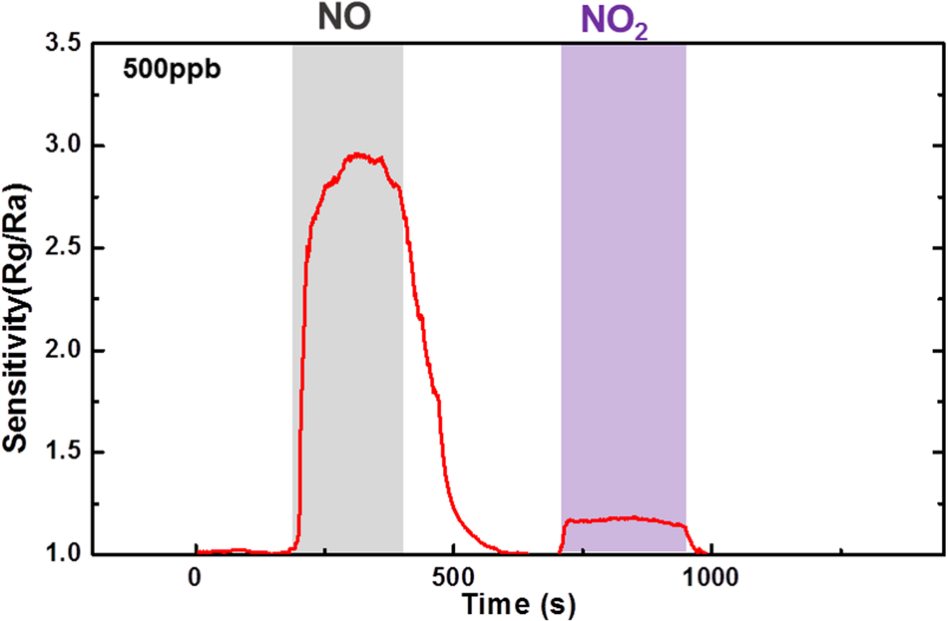
Sensitivity of ZGO gas sensor for NO and NO_2_ with 500 ppb at 300 °C.

Figure 5 illustrates the transient response of ZGO gas sensor with NO gas at 300 °C. The NO gas concentrations are 6250 ppb, 3125 ppb, 1000 ppb, 500 ppb, 250 ppb, and 125 ppb, respectively. As shown in the figure, the resistance increased on NO injection (gray region). NO gas molecules adsorbed onto ZnGa_2_O_4_ surface, and they captured electrons, leading to an increase in resistance. Figure 6 shows the sensitivity of ZGO gas sensor with 500 ppb of NO and NO_2_. It can be found that the ZGO gas sensor has a larger sensitivity of NO than that of NO_2_. The behavior can also support that the resistance increased abruptly and then decreased slowly as NO gas was injected to the chamber due to the NO gas transferring into NO_2_ in the air. The decrease in the resistance can possibly be ascribed to a decreasing in NO concentration, owing to the transformation of NO to NO_2_. By contrast, when NO gas was purged by fresh air (white region), the electrons returned to the conduction band of the ZGO thin film. Therefore, the resistance recovered to the original baseline. The sensitivity values are 22.21, 10.53, 5.03, 3.10, 2.01, and 1.88 with the NO concentrations of 6250 ppb, 3125 ppb, 1000 ppb, 500 ppb, 250 ppb, and 125 ppb, respectively.

**Figure 7:**
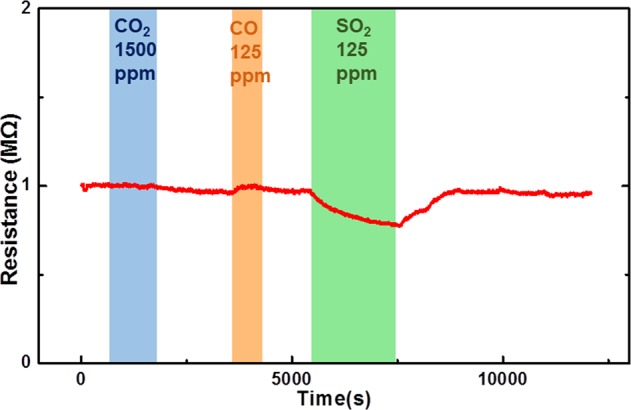
Transient response of ZGO gas sensor to CO_2_, CO, and SO_2_ with concentrations of 1500, 125, and 125 ppm, respectively.

**Figure 8:**
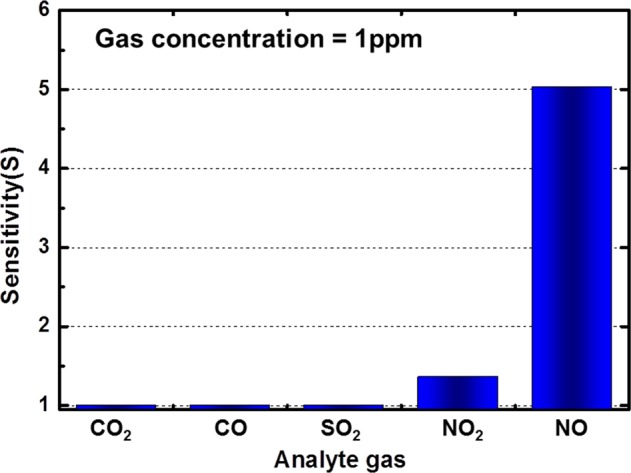
Sensitivity of ZGO gas sensor to CO_2_, CO, SO_2_, NO_2_, and NO at 300 °C.

To study the selectivity of the ZGO gas sensor, CO_2_, CO, and SO_2_ were injected with concentrations of 1500, 125 and 125 ppm at the same operating temperature (300 °C), respectively. Figure 7 shows the transient response of the sensor to those gases. The sensor hardly reacted with CO_2_ and CO. It did react with SO_2_, but it displayed a low sensitivity (1.27) against a high SO_2_ concentration (125 ppm). After comparing the gas concentration and the sensitivity, as shown in Fig. 8, the results imply that the ZGO gas sensor exhibits a high selectivity to NO at the operating temperature of 300 °C.

As concerning the mechanism of ZGO gas sensor being high responsivity for NO, it could be the fact that the dangling bonds on the surface of ZGO epilayer trapped oxygen molecules and turned them into adsorbed oxygen molecules. With different operating temperatures, the adsorbed oxygen molecules have different forms (O_2_^−^_(ads)_ or O^−^_(ads)_)^38^. The reactions of the adsorbed oxygen molecules are given by Eqs (2) and (3)^39^.

**Figure 9:**
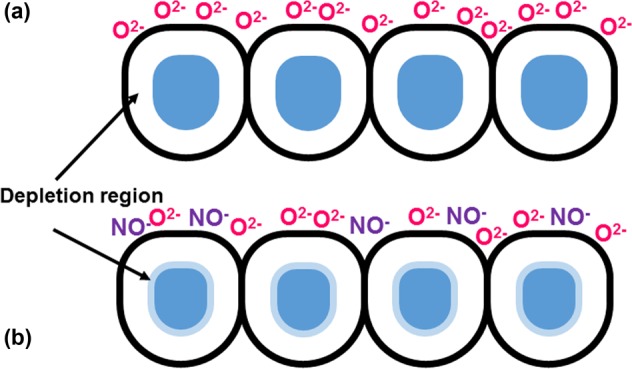
Interactions between the surface of ZGO thin film and adsorbed molecules (**a**) before injection of NO and (**b**) after injection of NO.

Figure 9(a) illustrates the interactions between the surface of the ZGO thin film and the adsorbed oxygen ions before NO gas injection. As mentioned above, the oxygen molecules dissociate and adsorb onto the ZGO surface of the thin film with the characteristic O_2_^−^ or O^−^ depending on the surface temperature. Both forms (O_2_^−^_(ads)_ or O^−^_(ads)_) extract electrons from the conduction band of the semiconductor, leading to the creation of the depletion region in the ZGO thin film. Figure 9(b) shows the interactions between the thin-film surface and the NO gas molecules. When NO gases were introduced into the chamber, NO gas molecules trapped the electrons due to high electronegative property and became NO^−^ which is shown in Eq. (4)^40^. On the other hand, NO gas molecules reacted with the adsorbed oxygen molecules as the Eq. (5) shows^40^. Both reactions further extracted the electrons, and that caused the conductivity to decrease. As shown in Fig. 3(a), there is an obvious enhancement on sensitivity to NO as operating temperature increases from 100 °C to 150 °C (red arrow). This is due to the fact that high temperature makes the particles originally adsorbed on the surface desorb which allows more states on ZGO surface to react with NO gas molecules. Furthermore, high temperature also changes the form of adsorbed oxygen molecules. As the temperature is low, O_2_^−^ is the dominant adsorbed oxygen molecule. When the temperature ramps up, the dominant molecule becomes to O^−^ which is more reactive. This makes NO gas molecules more easily react with adsorbed oxygen molecules and increase the sensitivity. On the other hand, high temperature provides more kinetic energy for gas molecules to move in the chamber, and also speeds up the process of reactions between NO gas molecules and adsorbed oxygen molecules. This resulted in a dramatically reducing on response time and recovery time as operating temperature increasing.

2 $${{\rm{O}}}_{2(\mathrm{gas})}+{{\rm{e}}}^{-}\to {{\rm{O}}}_{2({\rm{ads}})}^{-}$$

3 $${{\rm{O}}}_{2({\rm{a}}{\rm{d}}{\rm{s}})}^{-}+{{\rm{e}}}^{-}\to 2{{\rm{O}}}_{({\rm{a}}{\rm{d}}{\rm{s}})}^{-}$$

4 $${{\rm{NO}}}_{(\mathrm{gas})}+{{\rm{e}}}^{-}\to {{\rm{NO}}}_{({\rm{ads}})}^{-}$$

5 $${{\rm{N}}{\rm{O}}}_{({\rm{g}}{\rm{a}}{\rm{s}})}+{{\rm{O}}}_{2({\rm{a}}{\rm{d}}{\rm{s}})}^{-}/{{\rm{O}}}_{({\rm{a}}{\rm{d}}{\rm{s}})}^{-}\to {{\rm{N}}{\rm{O}}}_{({\rm{a}}{\rm{d}}{\rm{s}})}^{-}+{{\rm{O}}}_{2({\rm{g}}{\rm{a}}{\rm{s}})}/{{\rm{N}}{\rm{O}}}_{2({\rm{a}}{\rm{d}}{\rm{s}})}^{-}$$

Table 1 presents the response time and recovery time of the ZGO gas sensor for different concentrations of NO gas at the operating temperature of 300 °C. The results imply that the sensor rapidly detected NO gas. The response times to all gas concentrations were shorter than 60 s, and no relation was observed between the NO gas concentration and response time. The response time of 125, 250, and 500 ppb are almost the same. This implies that NO gas molecules could adsorb onto ZGO thin film surface very easily in this concentration range. However, as the gas concentration increases (>1000 ppb), there are more and more molecules try to adsorb onto the ZGO surface. The molecules have to spend more time to find the unoccupied states on the surface. Therefore, the response time increases. By contrast, it was found that the recovery time increased with decreasing gas concentration. This could possibly be ascribed to the fact that the surface completely absorbed the NO under high concentration. Owing to purging by fresh air, the gas sensor surface desorbed NO immediately. The recovery time was related to the concentration difference (reference is the background concentration). The recovery time was shorter when the concentration difference was high.

**Table 1:** Response time and recovery time of the ZGO gas sensor to NO with different gas concentrations at 300 °C.

NO (ppb)	6250	3125	1000	500	250	125
Resposne time (s)	57	59	54	35	38	36
Recovery time(s)	78	84	90	167	185	208

**Figure 10:**
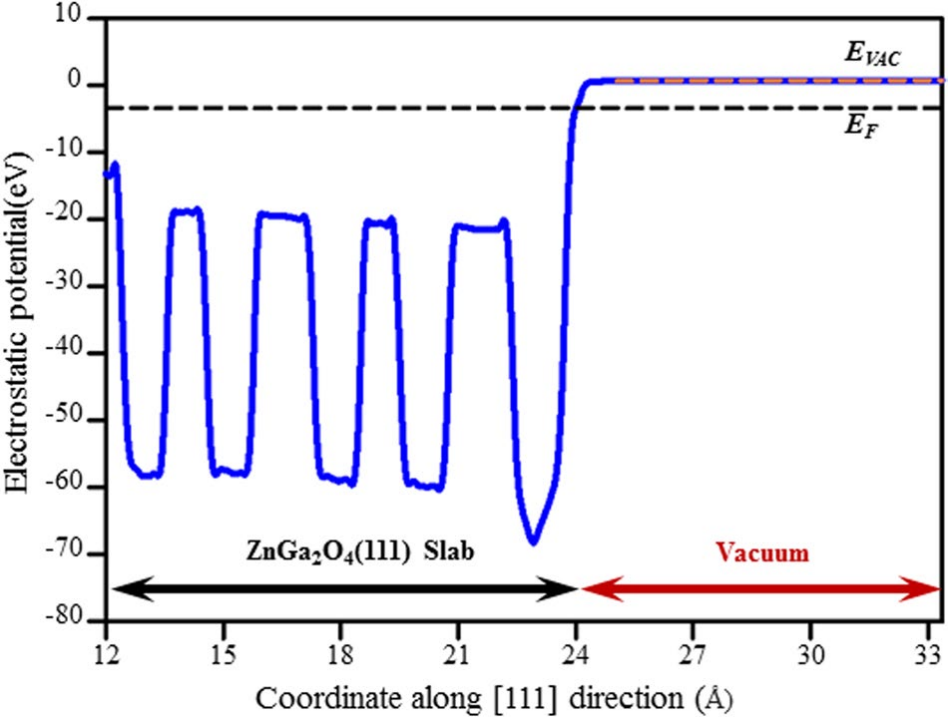
Planar average (solid line) and vacuum level E_VAC_ and Fermi level E_F_ (dashed lines) of electrostatic potential near Ga-Zn-O-terminated ZGO(111) surface computed within DFT-GGA functional.

The work functions of the clean ZGO (111) surface and the adsorption bonding of NO on the ZGO (111) surface are summarized in Table 2. The work function of the clean ZGO(111) surface is 4.04 eV, and it was used as a reference for the work function change calculations herein. Figure 10 shows the energy diagram of Model N-Ga, which in turn shows the work function, 4.15 eV, between the vacuum level E_VAC_ and the Fermi level E_F_. The work function changes in the cases of Models N-Ga and N-Zn were 0.11 and 0.04 eV, respectively, indicating a more sensitive adsorption site of atomic Ga on the ZGO(111) surface. For two NO molecules, we verified that the work function changes of the models in terms of magnitude follows the order: Model 2N-Ga (0.26 eV) > Model 2N-Ga-Zn (0.23 eV) > Model 2N-Zn (0.17 eV). This ordering remarkably demonstrates that high concentrations of NO gas exhibit high selective gas adsorption for NO onto ZGO thin film.

**Table 2:** Work functions of clean ZGO(111) surface and adsorption bonding of NO on ZGO(111) surface.

Models	*E*_*VAC*_ (eV)	*E*_*F*_ (eV)	*Φ* (eV)	Δ*Φ* (eV)
ZnGa_2_O_4_(111)	0.66	−3.38	4.04	—
N-Ga	0.79	−3.36	4.15	0.11
N-Zn	0.73	−3.35	4.08	0.04
2N-Ga	0.96	−3.34	4.30	0.26
2N-Ga-Zn	0.88	−3.39	4.27	0.23
2N-Zn	0.65	−3.56	4.21	0.17

## Conclusion

A NO gas sensor based on a ZGO epilayer grown by MOCVD was investigated in this work. The results indicated that ZGO gas sensor exhibited high sensitivity, reversibility, and selectivity in detecting NO at the operating temperature of 300 °C. When exposed to 125 ppb NO, a sensitivity of 1.88 was observed. The response time and recovery time were 36 and 208 s, respectively. The sensor has high sensitivity to NO, but it hardly reacts with CO_2_, CO, and SO_2_. Besides, ZGO also shows a larger response to NO than to NO_2_. Moreover, the results of a first-principles simulation proved that the ZGO gas sensor exhibits a great response to NO gas because of the large change in work function when NO gas molecules are adsorbed onto the ZGO thin-film surface. The above results prove that the proposed ZGO thin film gas sensor has the potential for use in IOT applications.

## Methods

**Figure 11:**
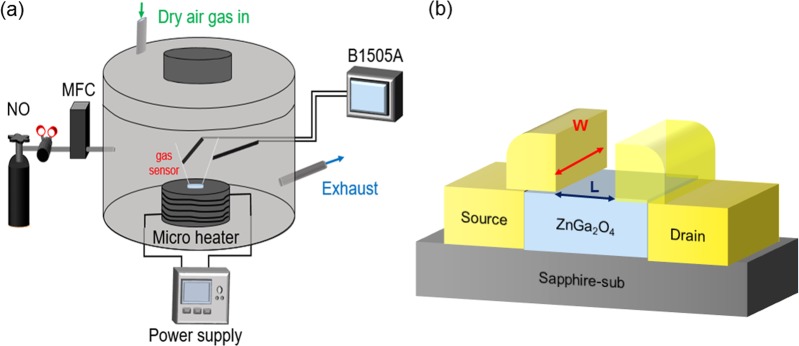
(**a**) Schematic of ZGO gas sensor measurement system. (**b**) Device structure of ZGO gas sensor, where L = 30 um and W = 250 um.

ZGO thin films with a thickness of 100 nm were grown on a c-plane (0001) sapphire substrate at 600 °C by MOCVD. The precursors of Zn and Ga are diethylzinc (DEZn) and triethylgallium (TEGa), respectively. Purified Ar and oxygen were employed as the carrier gas and oxide source, respectively. The thickness of epilayer was approximately 100 nm under a growth rate of 0.8 nm/min. After epilayer growth, the process commenced with mesa isolation in an induced coupled plasma etching system by BCl_3_/Cl_2_/Ar. The mesa isolation process etched the epilayer onto the sapphire substrate and left the plateau. The electrodes were composed of Ti/Al/Ni (50/75/25 nm) multilayer metals deposited using an E-gun evaporator, and they were patterned by a lift-off process. The channel length *L* and width *W* were 30 and 250 µm, respectively. After the completion of the above mentioned processes, the device was annealed under 700 °C for 1 h. A schematic of the gas sensor measurement system and the device structure are shown in Fig. 11.

Regarding the sensing characteristics of the gas sensor, sensitivity and response time are important parameters. Sensitivity can be defined as Ra/Rg for reducing gases and Rg/Ra for oxidizing gases, where Ra and Rg denoted as the resistance of the gas sensor with dry air and that to the target gases, respectively^41^.

Response time and recovery time are defined as the time required for the sensor to reach 90% of its steady resistance and back to 10% of the value^42^.

**Figure 12:**
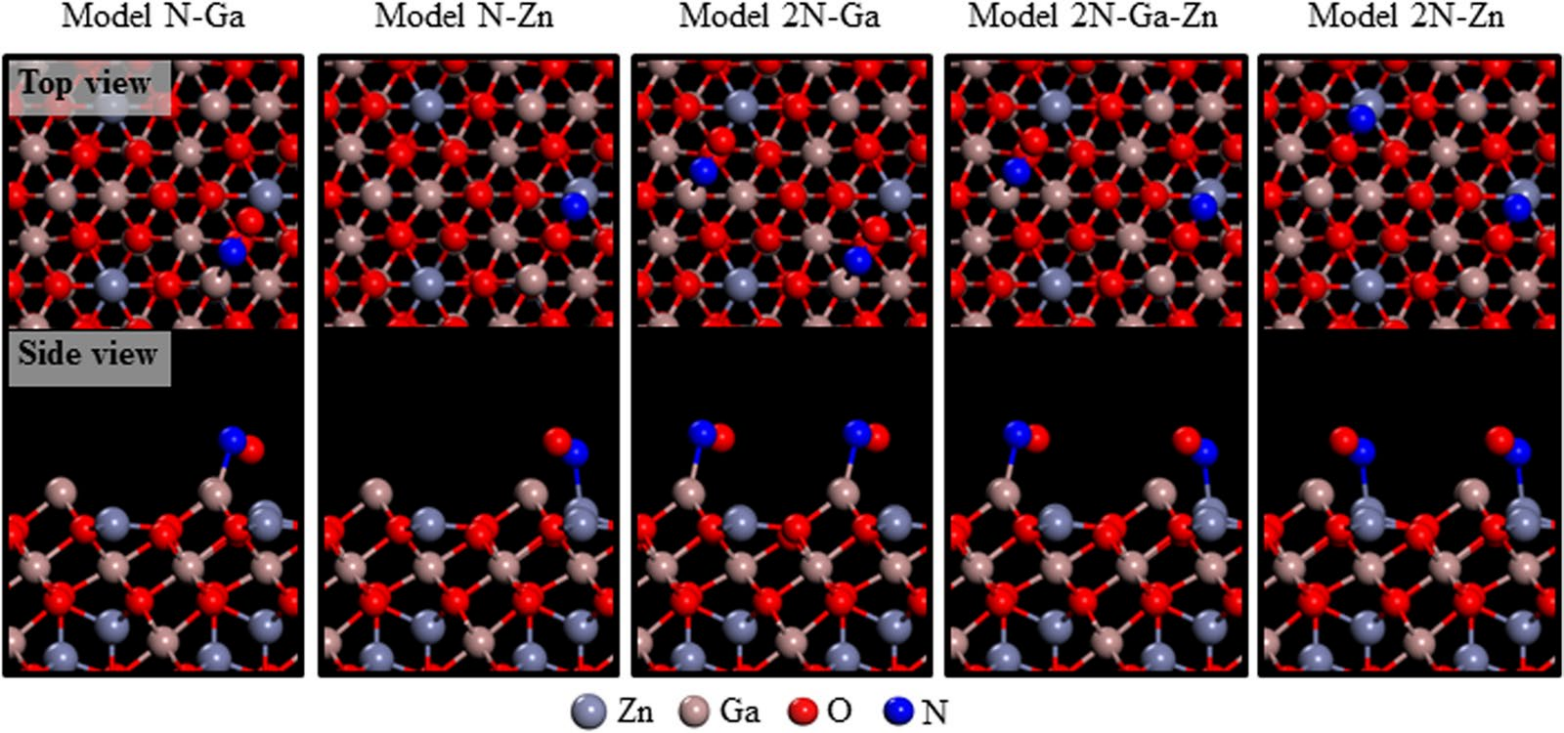
Atomistic representations of detailed N-Ga and/or N-Zn bonding arrangements pertaining to NO exposure on Ga-Zn-O-terminated ZGO(111) models. The atoms are represented by spheres: Zn (gray), Ga (brown), O (red), and N (blue).

To study the mechanism of the reaction between NO gas and ZGO, the reactions of the gas with different surface structures were simulated by first-principle calculations. In general, the sensor response is typically characterized by work function changes in gas-sensitive materials. If we assume that the gap between the conduction band and the Fermi level in the bulk is not affected by gas adsorption at the surface, the work function changes because of the adsorption process of oxidizing gases as opposed to those caused by the clean surface can be written as follows^43^.

6 $${\rm{\Delta }}{\rm{\Phi }}={\rm{\Delta }}{\rm{\chi }}+k{\rm{T}}\,{\rm{In}}\frac{Rg}{Ra},$$

where Δχ denotes changes in electron affinity, and the second term corresponds to changes in band bending. Here, k and T are Boltzmann constant and temperature, respectively. Equation (6) shows that the work function changes can be described in terms of sensitivity (Rg/Ra for oxidizing gases). Moreover, we present *ab initio* simulations of NO adsorption behavior onto ZGO (111) thin film to elucidate the sensitivity of our gas sensor. Our simulations were based on the density functional theory (DFT), as implemented in the Vienna ab initio simulation package code^44–46^. The projector-augmented wave method and the generalized gradient approximation (GGA) with the Perdew-Wang (PW91) exchange-correlation functional were employed to efficiently treat ion-electron interactions^47,48^. The electronic configurations of the valence electrons were N: 2 s2/2p3, O: 2s2/2p4, Zn: 4s2/3d10, and Ga: 4s2/4p1. The ZGO (space group: 227 Fd-3 m) alloy and NO gas (space group: 99 P4mm) were constructed using the bulk crystalline and the gas configurations, respectively. In the ZGO(111) surface slab models, we adopted a $\sqrt{2}\times \sqrt{2}$ basal setting (11.85 Å × 11.85 Å) for all adsorption calculations. The repeated slab geometry layers fixed at Zn_16_Ga_32_O_64_ were separated by vacuum regions equivalent to a thickness of 20 Å. Ga-Zn-O-terminated ZGO (111) surfaces are preferred with a low surface energy of 0.096 eV/Å^2^, and therefore, such surfaces were adopted in the present work^49^. Reactions of NO molecules on Ga-Zn-O-terminated ZnGa_2_O_4_(111) surfaces were modeled to calculate the work function changes or the NO sensitivity. The Brillouin zones were created using a 3 × 3 × 1 Gamma-Center grid and a 400-eV energy cutoff in the surface reaction models to obtain the optimized adsorption bonding of NO molecules on Ga-Zn-O-terminated ZnGa_2_O_4_(111) surfaces (Fig. 12). As one NO molecule approached the Ga-Zn-O-terminated ZnGa_2_O_4_(111) surface, the nitrogen of NO bonded with the gallium atom on the ZGO (111) surface shown in Model N-Ga. In Model N-Zn, the nitrogen of NO bonded with the zinc atom on the ZGO (111) surface. To compare the concentrations of NO, we constructed Model 2N-Ga, which showed that each of nitrogen of two NO molecules was bonded to the gallium atoms on the ZGO (111) surface. In Model 2N-Ga-Zn, each nitrogen of two NO molecules were bonded to one zinc atom and one gallium atom on the ZGO (111) surface. In Model 2N-Zn, each nitrogen of two NO molecules were bonded to the zinc atoms on the ZGO (111) surface.
